# A hypothesis-driven pulmonary–cerebral mechanistic framework of neurological decompression illness in repetitive breath-hold diving

**DOI:** 10.3389/fphys.2026.1911448

**Published:** 2026-09-11

**Authors:** Kiyotaka Kohshi, Hideki Tamaki, Yoshitaka Morimatsu, Tatsuya Ishitake, Frédéric Lemaître

**Affiliations:** 1 Division of Neurosurgery, Nishinihon Hospital, Kumamoto, Japan; 2 Department of Environmental Medicine, Kurume University School of Medicine, Kurume, Japan; 3 Division of Surgery and General Medicine, Tamaki Hospital, Yamaguchi, Japan; 4 Faculty of Sport Sciences, University of Rouen, Mont-Saint-Aignan, France; 5 CRIOBE USR 3278, CNRS-EPHE-UPVD, PSL, Paris, France

**Keywords:** arterial gas embolism, decompression sickness, intrapulmonary arteriovenous anastomoses, MRI, nitrogen bubble, vasogenic edema

## Abstract

This hypothesis-driven narrative review synthesizes physiological, clinical, and neuroimaging evidence to advance an integrated pulmonary–cerebral framework for decompression illness (DCI) in repetitive breath-hold diving. Three unresolved features are addressed: the predominance of cerebral involvement, the characteristic localization of lesions to external watershed and distal arterial territories, and the gradual evolution of neurological deficits. Drawing on converging evidence from pulmonary vascular physiology and cerebral gas kinetics, we propose that venous nitrogen bubbles generated during repetitive dives intermittently bypass pulmonary filtration through transient recruitment of intrapulmonary arteriovenous anastomoses (IPAVA), providing a plausible route for episodic arterialization. Once lodged in distal cerebral arteries, these bubbles may enlarge through diffusion of nitrogen from supersaturated brain tissue, potentially contributing to progressive vascular obstruction, endothelial activation, blood–brain barrier disruption, and vasogenic edema. This review articulates a hypothesis-generating integrative framework in which IPAVA-mediated arterialization and diffusion-driven bubble enlargement may together provide a plausible physiological interpretation for the progressive, watershed-predominant cerebral injury unique to repetitive breath-hold diving. Although direct evidence remains limited—particularly regarding dynamic IPAVA behavior and quantitative nitrogen kinetics— the available data appear broadly compatible with a proposed mechanism centered on episodic arterialization followed by diffusion-driven bubble growth. By framing breath-hold diving–related DCI as a coupled pulmonary–cerebral process, this review proposes a testable conceptual foundation for future integrative research aimed at clarifying IPAVA dynamics, cerebral microvascular vulnerability, and nitrogen transport within supersaturated tissues.

## Introduction

Breath-hold diving is practiced worldwide for subsistence, commercial harvesting, and recreation. Traditional communities such as the Japanese Ama routinely perform hundreds of dives per day to depths of 10–20 meters with minimal surface intervals (Mohri et al., 1995; Tamaki et al., 2010a; Lemaître et al., 2014). Although these dives do not involve compressed-gas exposure, repetitive breath-hold diving has long been associated with neurological symptoms resembling decompression illness (DCI).

Despite decades of investigation, no existing framework has fully and consistently integrated the pulmonary, cerebral, and temporal characteristics of this condition. Breath-hold diving–related DCI is now recognized as a distinct clinical entity incorporating features of both decompression sickness (DCS) and cerebral arterial gas embolism (CAGE) (Barak et al., 2020; Kohshi et al., 2021; Blogg et al., 2023). Neurological symptoms predominate, spinal involvement is uncommon (Wong, 2006), and deficits typically evolve gradually rather than presenting abruptly (Kohshi et al., 2021).

Two central questions remain unresolved:

1. why extensive cerebral lesions develop despite relatively small detectable bubble loads (Lemaître et al., 2014; Schipke and Tetzlaff, 2016; Barak et al., 2020), and
2. why neurological symptoms progress over several hours rather than presenting suddenly (Kohshi et al., 2021).

Although individual studies have described clinical patterns, pulmonary shunting physiology, or neuroimaging findings, no prior review has integrated these domains into a unified mechanistic framework.

This narrative review aims to bridge this gap by synthesizing clinical, physiological, and radiological evidence to propose a coherent pulmonary–cerebral model explaining the distinctive neurological phenotype of repetitive breath-hold diving.

## Methods

A narrative review approach was adopted to integrate clinical, physiological, and radiological evidence relevant to neurological DCI in repetitive breath-hold diving. To ensure comprehensive coverage of the literature, a broad but structured search strategy was used. A PubMed and Google Scholar search (up to December 2025) was conducted using the following Boolean combination: (“breath-hold diver” OR “breath-hold dives” OR “breath-hold diving” OR “breath-hold”) AND (“decompression illness” OR “decompression sickness” OR “taravana” OR “cerebral infarction”). This search yielded 92 publications, all of which were manually screened. This strategy was selected because neurological symptoms and MRI findings are often not included in titles or abstracts—particularly in older reports—and more restrictive filters would exclude clinically important cases.

Given the small number of published cases, heterogeneity in study methodologies, and absence of quantitative physiological datasets, a narrative, integrative synthesis was prioritized over formal systematic analysis. Emphasis was placed on recurring clinical patterns, convergent physiological mechanisms, and characteristic neuroimaging features. Thirteen cases across ten publications met inclusion criteria, with one duplicate removed.

Therefore, this review adopts a narrative approach not to quantitatively synthesize evidence, but to integrate heterogeneous clinical, physiological, and radiological findings into a testable pathophysiological model.

## Results

### Clinical observations

The clinical characteristics of the 12 included cases are summarized in **Table 1**. These cases collectively illustrate the distinctive neurological profile associated with repetitive breath-hold diving.

**Table 1:** Summary of cases with neurological decompression illness in repetitive breath-hold diving.

No	Age/gender	Dive depth (m)	Dive time (sec)	Surface interval (min)	Diving duration (hr)	Neurological symptoms	Timing of MRI	FLAIR/ DWI/ADC	R-L shunt
1	44/M (Kohshi et al., 1998)	15-25	60	1-3	3.5	Speech difficulty, R hemiparesis, R sensory loss	14 days	H/NR/NR	NR
2	38/M (Kohshi et al., 1998)	15-25	60	1-3	4	L hemiparesis, sensory disturbance, LOC	4 years	H/NR/NR	NR
3	33/M (Kohshi et al., 2000)	15-25	60-90	1	4-5, lunch break	Dizziness, R homonymous hemianopsia	4^th^ day	H/NR/NR	No
4	39/M (Kohshi et al., 2000)	20-23	60-90	1	4-5, lunch break	Dizziness, R weakness and numbness	3^rd^ day	H/NR/NR	No
5	34/M (Tamaki et al., 2010)	22	40-80	<0.5	2	R paresthesia, dizziness, diplopia	Next day	H/NR/NR	NR
6	57/M (Cortegiani et al., 2013)	30-35	130-170	1-1.5	2.5	Dizziness, blurred vision, numbness, seizure	One day	(T1-image)	No
7	NR/NR (Matsuo et al., 2014)	25-30	NR	NR	6	Dizziness, gait instability, L quadrantanopia	On admission	H/H/H	NR
8	38/M (Accurso et al., 2018)	40	<84	NR	4	L leg weakness and paresthesia	2 days	H/NR/NR	No
9	39/M (Guerreiro et al., 2018)	30	120	short	5	Aphasia	NR	H/H/H	NR
10	65/M (Kohshi et al., 2020)	10-20	NR	NR	4	Slurred speech, R-hand paresthesia, unsteady gait	2 hours	H/H/L	NR
11	31/M (Sanchez-Villalobos et al., 2022)	~30	>120	<2	3	R weakness, paresthesia, confusion	4 days	H/H/H	No
12	45/M (Druelle et al., 2024)	10-41	99-207	4-6	0.5-1	LOC, seizure	1 day	H/N/H	No

Parentheses: published years and references, MRI, magnetic resonance imaging; FLAIR, fluid attenuated inversion recovery; DWI, diffusion-weighted imaging; ADC, apparent diffusion coefficient, R/L, right/left; M, male; H/N/L, high/normal/low; NR, not reported in original publication; LOC, loss of consciousness.

Neurological complications represent the predominant clinical concern in breath-hold divers, with cerebral involvement occurring far more frequently than non-neurological manifestations (Wong, 2006; Blogg et al., 2023). Although both breath-hold and compressed-air divers may experience stroke-like events due to arterial gas embolism, the neurological disorders in breath-hold divers typically evolve gradually rather than presenting with the abrupt, severe onset characteristic of classical CAGE (Kohshi et al., 2021).

A survey of 173 Ama divers identified twelve individuals with a history of stroke-like episodes, particularly among those performing deeper dives with short surface intervals (Tamaki et al., 2010a). Reported symptoms included sensory deficits, motor weakness, speech disturbances, visual impairment, and dizziness, whereas limb pain and pulmonary symptoms—common in compressed-air DCI (Mitchell, 2024)—were uncommon, underscoring a distinct clinical profile.

A defining feature is the temporal progression of deficits. Neurological deterioration typically emerges within 24 hours of diving and often worsens over several hours (Kohshi et al., 2021), a pattern resembling ultra-early neurological deterioration (UND) in acute ischemic stroke (Seners et al., 2015). Five of six experienced Ama divers in prior reports exhibited UND-like progression, with deficits developing or worsening hours after repetitive dives (Kohshi et al., 1998, 2000, 2020; Tamaki et al., 2010b). Similar trajectories were described in Paulev’s classic report (1965) and in several recent cases (Cortegiani et al., 2013; Accurso et al., 2018; Sánchez-Villalobos et al., 2022; Druelle et al., 2024).

Loss of consciousness—common in compressed-air CAGE (Mitchell, 2024)—is rare in breath-hold divers (Blogg et al., 2023). Instead, deficits usually begin mildly and progress gradually, supporting a mechanism of progressive vascular compromise rather than sudden, massive arterial obstruction.

### Neuroradiological findings

Brain MRI in breath-hold divers with neurological DCI reveals three recurring features:

1. Lesions predominantly located in external watershed areas and distal arterial territories
2. Imaging characteristics consistent with bubble-induced edema
3. Mixed cytotoxic–vasogenic patterns resembling those observed in other forms of CAGE

MRI studies in Ama divers demonstrate single or multiple lesions corresponding to clinical deficits, distributed across the cortex, subcortex, basal ganglia, brainstem, and cerebellum (Kohshi et al., 1998, 2000, 2020; Tamaki et al., 2010b; Matsuo et al., 2014). The predominance of external watershed and distal branch lesions is most consistent with an embolic mechanism, as wedge-shaped watershed infarcts are classically associated with embolic occlusion (Mangla et al., 2011). Lesions in perforating artery territories—sometimes attributed to intrinsic small-vessel disease—may also arise from microembolic events (Horowitz et al., 1992).

Diffusion-weighted imaging (DWI) and apparent diffusion coefficient (ADC) mapping have improved differentiation between cytotoxic and vasogenic edema (Ho et al., 2012). In several breath-hold divers, MRI obtained one day or more after symptom onset showed slight DWI hyperintensity without ADC reduction, resolving within weeks—findings consistent with vasogenic edema (Matsuo et al., 2014; Guerreiro et al., 2018; Sánchez-Villalobos et al., 2022; Druelle et al., 2024). In contrast, one Ama diver imaged within two hours of symptom onset exhibited DWI hyperintensity with decreased ADC, indicating acute ischemia (Kohshi et al., 2020). These observations suggest that cerebral injury may begin with ischemia in the hyperacute stage and subsequently evolve into vasogenic edema, reflecting both vascular obstruction and secondary endothelial responses.

MRI findings in non-diving-related CAGE typically show early cytotoxic edema followed by vasogenic edema (Kang et al., 2019; Oka et al., 2020; Timpone and Callen, 2024), a pattern mirrored in breath-hold diving–related DCI. Some cases have been described as resembling posterior reversible encephalopathy syndrome (PRES) (Matsuo et al., 2014; Sánchez-Villalobos et al., 2022; Druelle et al., 2024), but the distribution differs: PRES is typically symmetric and parieto-occipital (Tetsuka and Ogawa, 2019), whereas breath-hold diving lesions are asymmetric and watershed-predominant.

Taken together, clinical and imaging evidence appears compatible with a pathophysiological process involving distal arterial embolization, progressive vascular compromise, and evolving edema, which may plausibly arise from with arterialized venous bubbles that could undergo secondary enlargement within distal cerebral arteries.

## Discussion

### Integrated mechanistic interpretation

**Figure 1:**
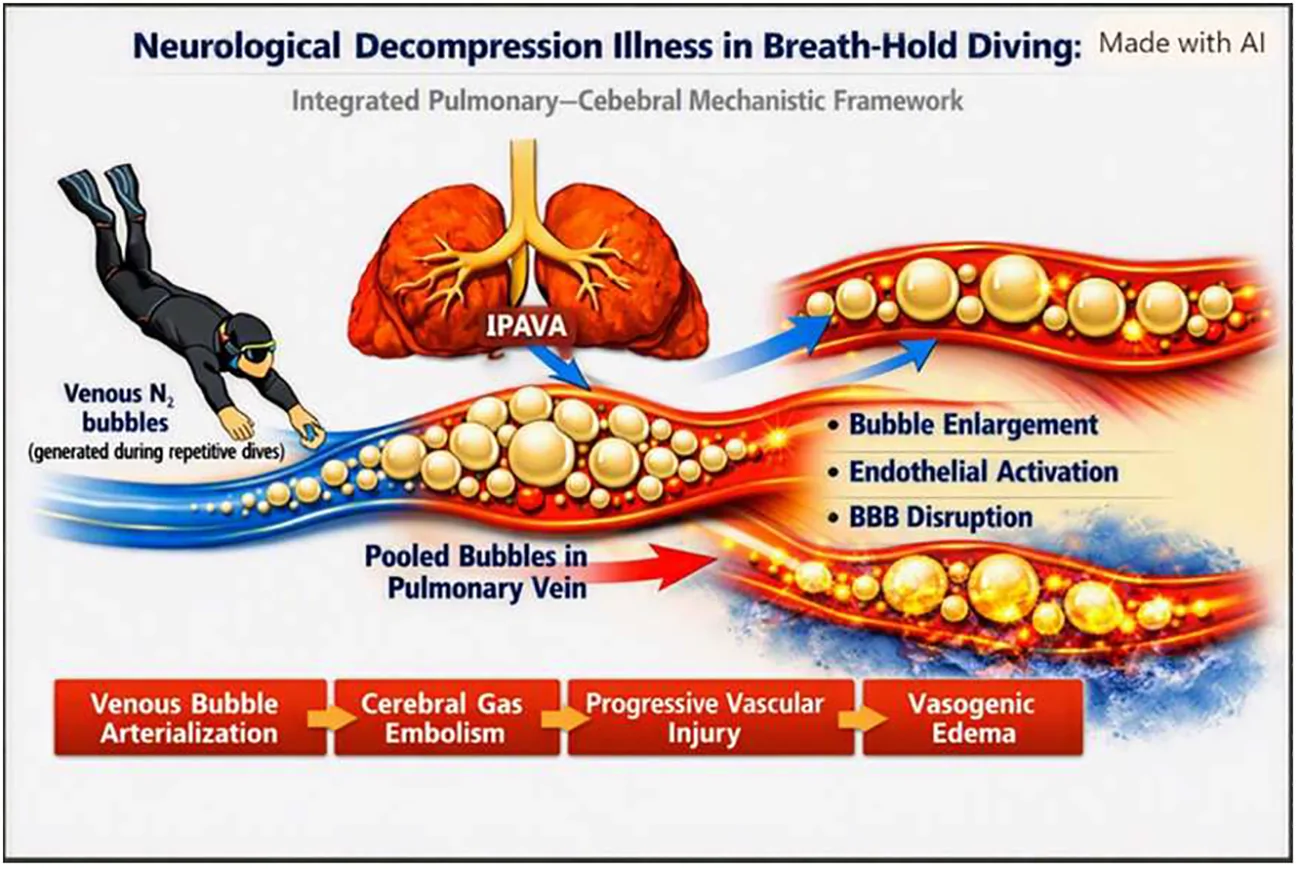
Integrated pulmonary–cerebral mechanistic framework of neurological decompression illness in repetitive breath-hold diving. Venous nitrogen (N_2_) bubbles generated during repetitive dives may bypass pulmonary filtration through transient distension of intrapulmonary arteriovenous anastomoses (IPAVA), allowing episodic release of pooled bubbles into the arterial circulation. Arterialized bubbles lodge in distal cerebral arteries, where diffusion-driven bubble enlargement and endothelial activation may lead to blood–brain barrier disruption and vasogenic edema. The schematic illustrates the continuum from venous bubble formation to progressive cerebral microvascular injury.

Neurological DCI in repetitive breath-hold diving may be interpreted as a plausible physiological continuum, although the mechanism remains hypothetical. The delayed symptom evolution, watershed-predominant lesions, and mixed cytotoxic–vasogenic edema suggest a dynamic process rather than a single catastrophic embolic event. Integrating pulmonary vascular behavior with cerebral gas kinetics may provide a coherent mechanistic interpretation for this distinctive phenotype (**Figure 1**).

**Figure 2:**
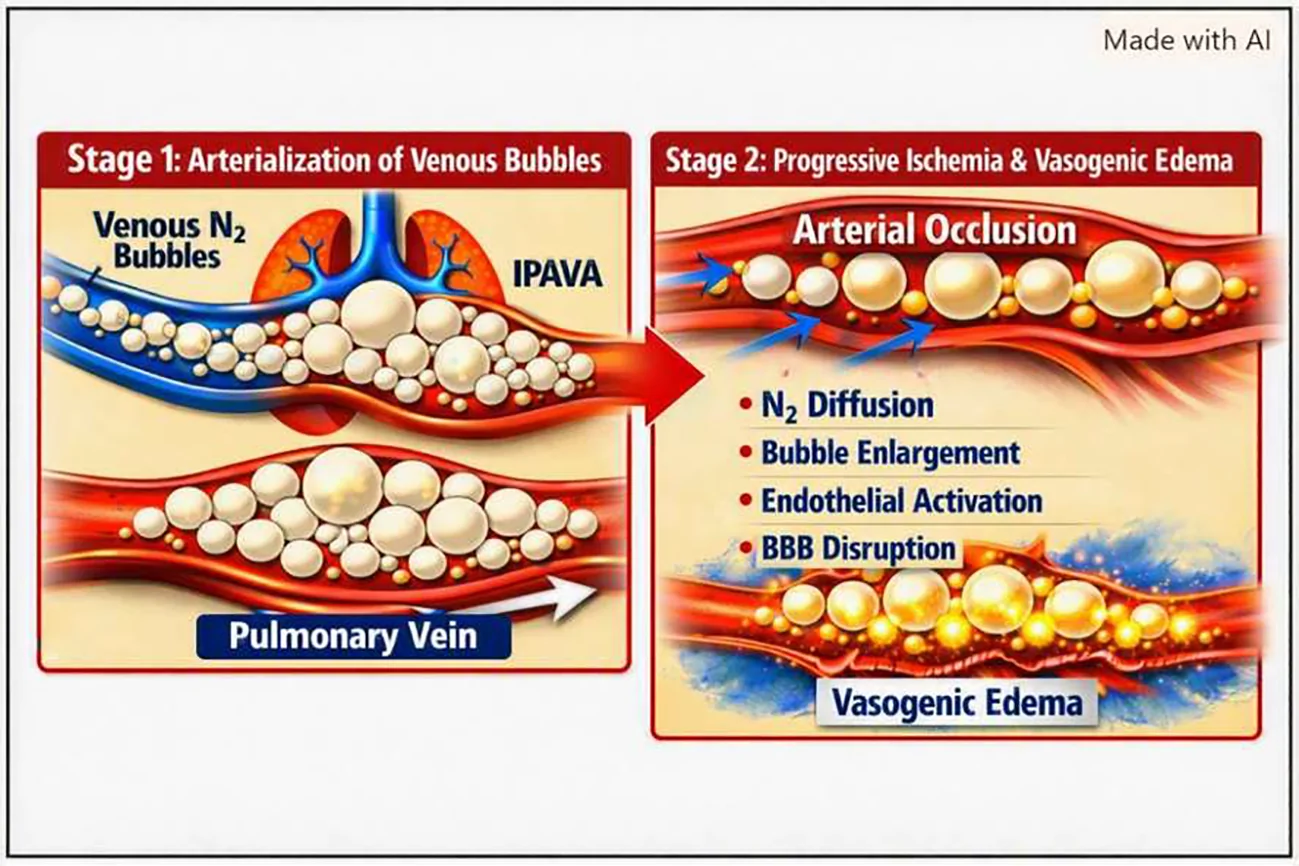
Schematic representation of the two-stage physiological model of neurological decompression illness in breath-hold diving. Stage 1: Intrapulmonary arteriovenous anastomoses (IPAVA)-mediated arterialization of venous bubbles. Venous nitrogen (N_2_) bubbles pass through IPAVA and enter the pulmonary veins, where accumulated or coalescing bubbles may form arterialized emboli. Stage 2: Diffusion-driven bubble enlargement and progressive ischemia. Once lodged in distal cerebral arteries, arterialized bubbles may enlarge through diffusion of N_2_ from supersaturated brain tissue, promoting endothelial activation, blood–brain barrier (BBB) disruption, and vasogenic edema. The schematic depicts the sequential vascular and tissue processes that may underlie progressive neurological injury.

The proposed model comprises two sequential but interdependent stages (**Figure 2**):

1. IPAVA-mediated arterialization of venous nitrogen bubbles, and
2. diffusion-driven enlargement of these bubbles within supersaturated distal cerebral arteries.

Together, these processes may help interpret the temporal progression and lesion distribution characteristic of breath-hold diving–related DCI.

### Pulmonary stage: IPAVA mediated arterialization

Repetitive breath-hold diving imposes unique cardiopulmonary stresses—central blood shift, hypoxia, and abrupt pressure transitions—that modulate the patency of intrapulmonary arteriovenous pathways. Anatomical substrates such as supernumerary arteries, “arterial loops,” and preterminal arterioles may form a parallel low-resistance vascular network with thin walls and minimal smooth muscle investment (Elliott and Reid, 1965; Tobin, 1966; Recavarren, 1966). These structures are highly distensible and capable of transiently accommodating particulate material, as suggested by early microsphere retention studies (Tobin, 1966). Although detailed anatomical confirmation is limited and the topic remains debated, several authors have suggested that supernumerary arteries, “arterial loops,” and preterminal arterioles may in fact represent the same vascular structures and constitute potential anatomical substrates of IPAVA (Eldridge et al., 2004; Dujić et al., 2005; Laurie et al., 2012).

During head-out immersion, simultaneous elevations in pulmonary arterial and venous pressures occur due to central blood pooling (Fitz-Clarke, 2018). Given the compliant architecture of IPAVA, such pressure elevations may promote passive distension of these pathways. Although direct visualization during breath-hold diving is lacking, the physiological plausibility of pressure-mediated IPAVA recruitment appears reasonable.

A key feature of this model is the episodic nature of arterialization (Schipke and Tetzlaff, 2016). Distended IPAVA may transiently trap venous nitrogen bubbles, although direct visualization of such retention or release has not yet been achieved. Once a critical volume accumulates—or when hypoxia and exercise during ascent further increase IPAVA patency (Lovering et al., 2015)— these pathways may, in principle, permit episodic forward flow, but this remains an inferred rather than demonstrated behavior. This hypothesized mechanism may help generate a plausible explanation for how relatively small detectable bubble loads can nonetheless result in significant cerebral embolization.

### Cerebral stage: diffusion driven bubble enlargement

Once arterialized bubbles enter the cerebral circulation, they tend to lodge in distal, low-flow territories such as external watershed zones, where nitrogen supersaturation is common. Repetitive breath-hold diving increases nitrogen loading in brain tissue (Olszowka and Rahn, 1987; Goldman and Solano-Altamirano, 2015), creating conditions that favor diffusion of nitrogen from supersaturated tissue into a trapped bubble (Tikuisis and Gerth, 2003).

The mathematical model developed by Goldman and Solano-Altamirano (2015) provides key quantitative insight: following five repetitive breath-hold dives to 100 feet, the nitrogen partial pressure in brain tissue decreases with a half-time of approximately 1.2 minutes after surfacing. During the dive, arterial nitrogen tension exceeds that of brain tissue; however, upon surfacing, arterial nitrogen tension falls rapidly, producing a reversal in which brain tissue becomes relatively nitrogen-rich. This reversal generates a steep diffusion gradient that drives nitrogen into any bubble lodged in distal cerebral arteries. The predicted nitrogen gradient reversal within the first few minutes after surfacing from repetitive breath-hold dives provides a physiologically plausible basis for early bubble enlargement. This mechanism may help explain why symptoms that are mild during the dive often begin to worsen within minutes of surfacing.

In low-flow territories, impaired washout prolongs bubble–tissue contact time, further amplifying diffusion-driven bubble growth. Enlarging bubbles trigger endothelial injury, leukocyte adhesion, complement activation, and microthrombosis (Barak et al., 2020; Marsh et al., 2023; Mitchell, 2024), ultimately leading to blood–brain barrier disruption and vasogenic edema. These dynamics appear compatible with the hypothesis that diffusion-driven bubble enlargement within supersaturated brain tissue contributes substantially to progressive cerebral injury (Cialoni et al., 2021; Vezzoli et al., 2024).

This cascade may be compatible with the characteristic clinical trajectory:

- mild symptoms during or immediately after diving
- progressive neurological deterioration over several hours
- MRI evolution from cytotoxic to vasogenic edema

This biphasic pattern of brain injury may be unique to breath-hold diving and is generally not observed in CAGE. The rapid post-surfacing nitrogen gradient predicted by the Goldman and Solano-Altamirano (2015) model may provide a quantitative physiological basis for the early worsening of neurological symptoms in breath-hold divers. These dynamics appear compatible with the hypothesis that diffusion-driven bubble enlargement within supersaturated brain tissue may contribute to the progressive cerebral injury associated with repetitive breath-hold diving.

Hypoxia is not considered a primary contributor to cerebral injury in breath-hold diving, and repetitive hypoxic exposure may even enhance ischemic tolerance (Patrician et al., 2021). Nevertheless, hypoxia-related physiological changes could act as minor modifiers of cerebrovascular conditions, and are therefore acknowledged only as secondary influences rather than central mechanisms (Kang et al., 2026).

### Integration of pulmonary and cerebral processes

Together, these proposed processes may account for the temporal progression and watershed-predominant lesion distribution characteristic of breath-hold diving–related DCI.

The pulmonary and cerebral stages are not independent phenomena but components of a single physiological continuum. IPAVA-mediated arterialization may provide the mechanism for bubble entry into the arterial circulation, whereas diffusion-driven enlargement within supersaturated brain tissue may explain delayed progression, evolving edema, and the mixed cytotoxic–vasogenic pattern observed on MRI.

Importantly:

- IPAVA recruitment alone cannot explain the clinical course
- diffusion-driven enlargement alone cannot explain the embolic distribution

Their combined effects may be compatible with the observed pattern of injury.

Although cardiac and systemic circulatory factors are not primary drivers in our model, they may modify bubble transit and cerebral perfusion. As venous bubbles pass through the heart and pulmonary circulation before reaching the brain, variations in cardiac output or pulmonary vascular pressure could influence arterialized bubble delivery. These factors are thus regarded as secondary modifiers rather than central mechanisms.

### Physiological context for the nanobubble hypothesis in breath-hold diving

The nanobubble hypothesis proposes that autochthonous nanobubbles form on hydrophobic surfaces and subsequently expand into obstructive bubbles within distal arteries (Arieli, 2019, 2023). While supported by ex vivo observations, *in vivo* evidence remains limited. Although nanobubbles may form wherever supersaturation and bubble nuclei coexist after decompression, several features of cerebral DCI may be difficult to reconcile with a mechanism based on nanobubble expansion within distal cerebral arteries. First, the clinical presentation of cerebral DCI is dominated by sudden, stroke-like neurological symptoms, which align more closely with transient arterialization of venous bubbles than with progressive intravascular nanobubble growth. Second, neuroimaging findings in breath-hold divers consistently show lesions in external watershed territories and arterial branch distributions, a pattern characteristic of embolic phenomena (Horowitz et al., 1992; Mangla et al., 2011). These MRI features suggest intermittent arterial inflow of bubbles rather than *de novo* bubble enlargement within the cerebral microcirculation, making a nanobubble-driven mechanism may be readily less compatible with the observed lesion topology.

In addition, the high cerebral blood flow and rapid nitrogen washout make sustained nanobubble growth in distal cerebral arteries physiologically unlikely. Nanobubbles may arise in any tissue after decompression, but the brain provides an environment in which such bubbles may tend to dissolve rather than expand.

In contrast, the inner ear—characterized by low perfusion and slow gas exchange—offers a milieu more favorable for nanobubble formation and growth (Arieli, 2019, 2023). The bubble-growth model of Goldman and Solano-Altamirano (2015), which predicts rapid bubble enlargement in the inner ear but not in the brain during repetitive breath-hold diving, is consistent with this interpretation.

Thus, while the nanobubble hypothesis remains conceptually attractive and cannot be excluded as a contributory mechanism in cerebral DCI, its physiological plausibility and clinical relevance may be greater for inner-ear DCI, where local conditions more readily support bubble expansion.

### Consideration of intracardiac shunting

Right-to-left shunting through a patent foramen ovale (PFO) is a recognized route for arterialization in compressed-gas diving (Mitchell, 2024), and a breath-hold diving case with coexisting PFO has been reported (Gempp and Blatteau, 2006). Thus, intracardiac shunting cannot be excluded as a potential contributor to cerebral gas embolization.

In the present review, six divers with neurological DCI underwent right-to-left shunt evaluation and none had a confirmed PFO, making it difficult to consider intracardiac shunting a primary mechanism in repetitive breath-hold diving. However, the fact that venous bubbles have been detected in the heart after breath-hold dives (Lemaître et al., 2014; Cialoni et al., 2016; Barak et al., 2020) suggests that PFO could become a more plausible contributing factor if more sensitive diagnostic methods were applied. Given that PFO is a well-recognized cause of paradoxical embolism, its potential role in neurological DCI among breath-hold divers should therefore be considered with appropriate caution.

Moreover, even if arterialization were to occur through a PFO, the subsequent cerebral injury would be expected to follow the same two-stage clinical progression proposed for IPAVA-mediated arterialization—initial symptoms followed by progressive neurological worsening over several hours—because the downstream cerebral processes would be identical regardless of the route of arterialization.

### Limitations

Evidence for dynamic IPAVA recruitment during breath-hold diving remains indirect, and neither episodic bubble release from distended pathways nor diffusion-driven bubble enlargement has been directly visualized. Quantitative *in vivo* data on nitrogen kinetics within occluded cerebral microvessels are also lacking, as current imaging modalities cannot capture bubble behavior in the pulmonary or cerebral microcirculation.

Clinical evidence is constrained by reliance on case reports and small series, with potential publication bias and heterogeneity in dive profiles, individual susceptibility, and imaging timing. These limitations complicate interpretation and underscore the need for integrative approaches combining contrast-enhanced echocardiography, advanced pulmonary vascular imaging, and computational modeling to test and refine the proposed framework.

Overall, the pulmonary–cerebral framework presented here should be regarded as a hypothesis-generating conceptual model, and its physiological plausibility requires future experimental and clinical validation.

This integrative interpretation is intended not as a definitive mechanism, but as a testable hypothesis that may guide future physiological and imaging-based investigations.

## Conclusions

Breath-hold diving–related DCI represents a distinct pathophysiological entity characterized by progressive, watershed-predominant cerebral injury. By integrating clinical observations with pulmonary shunting physiology and cerebral gas kinetics, this proposed two-stage framework may serve as a plausible integrative framework in which IPAVA-mediated arterialization of venous bubbles is followed by diffusion-driven enlargement within supersaturated brain tissue.

As the first review to explicitly integrate pulmonary and cerebral mechanisms, this framework may provide a conceptual foundation for future investigations, serving as a testable hypothesis rather than an established mechanism.

Accordingly, the proposed two-stage framework should be viewed as a hypothesis-generating model that invites further empirical evaluation.

## Physiological implications

The mechanisms proposed in this review may have broader physiological relevance extending well beyond breath-hold diving. Dynamic recruitment of IPAVA may contribute to transient right-to-left shunting in other hypoxic, exercise-induced, or high-stress states, with potential implications for systemic embolic load and pulmonary vascular regulation. Likewise, diffusion-driven bubble enlargement within supersaturated or endothelial-vulnerable tissues provides a conceptual framework for understanding microembolic injury in diverse environmental and clinical contexts. Together, these mechanisms underscore the importance of integrated cardiopulmonary–cerebral physiology when evaluating microvascular risk in extreme or physiologically stressed conditions.

## Future directions

Key priorities include:

1. Real-time assessment of IPAVA recruitment

Advances in contrast-enhanced echocardiography and pulmonary imaging may enable direct visualization of transient shunting.

2. Quantitative modeling of nitrogen kinetics

Computational models incorporating tissue supersaturation and bubble dynamics are needed to define conditions for progressive vascular obstruction.

3. Integrated pulmonary–cerebral physiological studies

Interdisciplinary approaches will be essential to validate and refine the proposed two-stage model.
